# Role of palliative radiotherapy in the management of mural cardiac metastases: who, when and how to treat? A case series of 10 patients

**DOI:** 10.1002/cam4.619

**Published:** 2016-02-16

**Authors:** Alireza Fotouhi Ghiam, Laura A. Dawson, Wael Abuzeid, Sarah Rauth, Raymond W. Jang, Eric Horlick, Andrea Bezjak

**Affiliations:** ^1^ Department of Radiation OncologyUniversity of TorontoTorontoOntarioCanada; ^2^ Radiation Medicine ProgramPrincess Margaret Cancer CentreUniversity Health NetworkTorontoOntarioCanada; ^3^ Division of CardiologyDepartment of MedicineUniversity Health NetworkUniversity of TorontoTorontoOntarioCanada; ^4^ Peel Regional Cancer CentreCredit Valley HospitalMississaugaOntarioCanada; ^5^ Department of Medical OncologyPrincess Margaret Cancer CentreUniversity Health NetworkUniversity of TorontoTorontoOntarioCanada

**Keywords:** Heart, heart neoplasms, neoplasm metastasis, palliative medicine, radiotherapy

## Abstract

Cardiac metastases (CM), although a rare manifestation of metastatic cancer, are increasing in incidence with the improved prognosis and increased longevity of many patients with cancer. This condition may be life‐threatening, especially for bulky rapidly growing tumors. Such cancer presentations may be amenable to palliative radiotherapy to improve symptoms and to prevent further cardiac function decline. Here, we report on our experience with 10 patients with mural CM who received radiotherapy (RT) to the heart with palliative intent. The radiation treatment was given in different clinical situations using different dose and fractionation, and with a variety of outcomes. Palliative RT was a reasonably effective treatment, leading to good radiographic response in five patients who were evaluable for radiologic response. The mean duration of response in responding patients was 6.3 months (range: 3–11 months). This report describing clinical dilemmas around CM radiation therapy summarizes the previous experiences with radiation in treatment of CM and may assist in the considerations of palliative treatment for these patients.

## Introduction

Cardiac metastases (CM) in patients with advanced malignancy are starting to be increasingly recognized, and their incidence has been increasing due to modern diagnostic tools, more effective cancer therapies, and an increase in patient's longevity 1, 2, 3, 4. The reported incidence of CM is variable, from 0.2% to 11.8% at autopsies 1, 2. Most of the CM are clinically silent and are overlooked until they reach an advanced stage impacting cardiac function, or are diagnosed only at postmortem 1, 2, 5, 6. They may manifest with nonspecific symptoms that are hard to distinguish from other causes of cardiovascular disease 5, such as right‐sided heart failure due to right‐sided obstructive tumors, pulmonary edema in left‐sided obstructive tumors, or secondary to systolic dysfunction from myocardial metastasis. They can also present with arrhythmia, chest pain, or life‐threatening conditions like outflow obstruction or cardiac tamponade 5, 7. A recognition of this condition is important as CM could potentially cause debilitating symptoms and even death if not treated 2, 8.

Despite significant advances in the treatment of metastatic malignancies, no standard‐of‐care therapy exists for managing the patients with CM, and considering generally poor outcome of these patients with oftentimes disseminated metastases 8, the decision to when and how to treat these patients is debatable. Patients are usually offered a constellation of different treatments, including repeated, palliative systemic treatment and/or surgical excision in rare cases 1, 2. Even though this condition is frequently associated with the terminal phase of a widespread disease, an appropriate palliative management may play a significant role in symptom control, improving quality of life, prevention of further complications, and possibly improving survival in selected cases 2, 8. The use of palliative radiotherapy (RT) in management of these patients has been limited presumably due to technical difficulties limiting the ability to safely deliver a clinically useful radiation dose to the heart and/or the concern about radiation‐induced toxicity.

To our knowledge, the published literature on palliative RT for malignant tumors metastatic to the heart is mainly limited to case reports (for summary see Table 1) and mostly reports on single cases. Here, we report on 10 cases with mural cardiac metastasis and describe the representative patients in different medical scenarios where RT was utilized with palliative intent. The aim of this study was to report on the experience gained in a large cancer centre and to review the literature with a view of understanding the role of palliative RT in the management of this potentially lethal condition.

**Table 1:** Summary of literature reporting patients with cardiac metastases treated with palliative RT where the details of radiation treatment are reported

Author	Number of cases	Primary	Location	Prior thoracic RT	Prior surgery	Dose (Gy)/fractionation	Response to RT	Duration of response	Comment
Al‐Mamgani et al. 4	1	Esophageal carcinoma	RV	Yes	No biopsy or surgery	20/5	Symptom improvement	2.5 months	Total RT dose of 45 Gy in 25 fractions; and an additional dose of 10–15 Gy through small portals
Orcurto et al. 6	1	Small‐cell lung cancer	RV	No	Biopsy	60 Gy	Asymptomatic patient	Not reported	
Chen et al. 7	1	Thyroid squamous cell carcinoma	RV	No	Biopsy	35/10	Decreased tumor size. Improved RVOT obstruction.	3 months	
Takenaka et al. 8	7	Soft‐tissue sarcoma	All heart chambers and pericardium	No	No biopsy or surgery	25/5, 45/15, 50/25, 60/30, 40/20, 32/16	MS of patients who received RT = 10.5 months (3.5 months: those who did not)	20 months (reported in only one case)	RT dose should be >45 Gy (immediately and prior to chemotherapy): RT ceased the need for continuous drainage of cardiac effusion
Cham et al. 9	38	Different histologies	Not reported	Not reported	Not reported	25–35 Gy in 3–4 weeks	Overall response rate: 61%	Breast cancer patients: 6 months (2–36 months)Other cancers: 1–4 months	RT was given with 250 kVp equipment using old techniques (clinical set‐up and portal films)
Dasgupta et al. 10	1	Anaplastic thyroid carcinoma	RARV	Yes	Biopsy	37.5/15	No change in size during RT; Decreased FDG uptake in post‐RT PET‐CT	2 months	Cardiac metastasis involved the pacemaker leads within the RA and RV. RT was given concurrently with Paclitaxel on days 1 and 8 of RT. No significant acute toxicity
Lemus et al. 18	2	Squamous cell cervical carcinoma	RV, Interventricular septum and RARV and interventricular septum	No	No biopsy or surgery	Case 1: 28.8/16Case 2: 60/30	Case 1: No responseCase 2: Not reported	Case 1: Patient died on treatmentCase2: Not reported (patient died 5 months after completion of RT)	Case 1: RT was delivered to the whole heart with Cisplatin given on the first day of RTCase 2: RT was given concurrently with infusional 5‐FU and Cisplatin
Magnuson et al. 20	1	Melanoma	LA and PV	No	No biopsy or surgery	45/25	Significant radiologic response	4 months	

MS, median survival; RV, right ventricle; RA, right atrium; LV, left ventricle; LA, left atrium; PV, pulmonary vein; RVOT, right ventricle outflow tract; RT, radiotherapy.

## Materials and Methods

The radiotherapy database at Princess Margaret Cancer Centre was searched to identify patients who received RT to the heart. Under an IRB approved retrospective review, the medical records were reviewed and details regarding the presentation, diagnosis, management, and follow‐up of these patients were obtained. Patients with primary malignant cardiac tumors, pericardial‐only metastasis, and malignant thrombi in inferior vena cava (IVC) or heart were excluded. During the 11‐year period between 2003 and 2014, ten patients with mural‐based metastatic deposits in the heart from various histologies were identified; given the different designations of tumor site and diagnosis, we suspect that this is not a comprehensive list of all patients with CM.

Clinical characteristics of these patients, the details of the tumor and radiation treatment are described in Table 2. Most patients had widespread metastatic disease and had received prior systemic therapy, in various combinations, or radiation in the past. Two patients were managed with surgery followed by postoperative RT to the tumor bed, and the remaining eight patients were treated with RT alone. Clinical symptomatic response and, if applicable, radiologic response to RT were evaluated. The duration of response was assessed as the time from the completion of RT to recurrence of the tumor mass, death without evidence of regrowth, or last follow‐up.

**Table 2:** The clinical features and treatment details of our 10 cases with mural cardiac metastases from different histologies who were treated with palliative RT

Patient	Age	Sex	Primary tumor	Tumor location	Prior surgery	Prior thoracic RT	Technique	Dose (Gy)	Fractions	Response	Duration of response (months)
1	51	Female	Myxoid liposarcoma	RV, LV	Yes	No	IMRT	45	25	Complete resolution of symptoms; Complete radiologic response	11 months
2	68	Male	Sarcoma	RA	Yes	Yes	IMRT	50	25	Complete resolution of symptoms	7 months
3	53	Female	Diffuse large B‐cell lymphoma	LV	No	No	POP AP/PA	25	10	Complete resolution of symptoms; partial radiologic response	3 months
4	74	Male	Adenocarcinoma of rectum	RV and Tricuspid valve	No	No	POP AP/PA	20	5	Partial resolution of symptoms	3 months
5	54	Male	Thymic carcinoma	RV	No	Yes	POP AP/PA	36	18	Partial resolution of symptoms; partial radiologic response	11 months
6	71	Male	Hepatocellular carcinoma	RV	No	No	VMAT	54	27	Stable symptoms; partial radiologic response	6 months
7	53	Female	Adenocarcinoma of lung	LA	No	No	POP AP/PA	20	5	Stable clinical symptom	3.5 months
8	67	Female	Thymoma	RA and SVC	No	Yes	3DCRT	30	20	Partial resolution of symptoms; partial radiologic response	6 months
9	29	Male	Adenocarcinoma of lung	LV	No	Yes	POP AP/PA	6	1	Patient died on treatment	
10	66	Male	Adenocarcinoma of rectum	RA	No	No	POP AP/PA	16	4	Patient died on treatment	

RT, radiation treatment; PORT, postoperative radiation treatment; LV, left ventricle, RV, right ventricle, LA, left atrium, RA, right atrium, SVC, superior vena cava; NA, not applicable; IMRT, intensity‐modulated radiation therapy; VMAT, volumetric modulated arc therapy; PCU, palliative care unit.

### Illustrative cases

Table 2 gives patient characteristics and treatment details of our 10 cases: eight are further described below. In addition, we also report on a case with cardiac metastasis and postmortem diagnosis of melanoma whose treatment was delayed because of inconclusive biopsy (patient 11).

### Postoperative RT with palliative intent to maximize local control and delay recurrence

*Patient 1* was a 51‐year‐old woman with metastatic myxoid liposarcoma from the left thigh. She presented with significant dyspnea and fatigue. CT imaging showed a 4.5 cm metastatic tumor involving the right and left ventricles, with pericardial extension. The cardiac metastasis progressed despite chemotherapy with Doxorubicin and Ifosfamide and was the only site of disease; the patient underwent a surgical resection, with close but negative margins. She received postoperative RT (45 Gy in 25 fractions) to the resection area to maximize local control. She had complete resolution of her respiratory symptoms (severe shortness of breath and orthopnea) and durable response till 7 months later when the follow‐up imaging showed multifocal metastases, but no sign of disease recurrence in the heart. The patient received chemotherapy. Four months later she showed a 3‐cm recurrent soft‐tissue mass involving the right ventricular wall and pericardium. A trial of chemotherapy was unsuccessful. Her symptoms progressed and she became oxygen‐dependent. Four months later, she passed away at home because of an extensive disseminated disease not amenable to any treatment.

### Palliative RT with successful symptom relief and reasonable response duration

*Patients 3, 4, and 5* had mural‐based CM from lymphoma, rectal cancer, and thymic carcinoma, respectively. They presented with different respiratory and general symptoms. The cardiac tumors were not amenable to surgical excision, and they received RT alone with palliative intent. RT was well tolerated in all cases with few acute side effects and resulted in complete or partial resolution of symptoms. Using simple techniques, the palliative RT was effective to relieve distressing symptoms of dyspnea, palpitation, and chest pain in these cases. The documented duration of response was between 3 and 11 months in these patients, and treatment was associated with limited toxicity.

### Pre‐emptive palliative RT to avoid/delay symptoms and/or local progression in asymptomatic or minimally symptomatic patients

*Patient 6* was a 71‐year‐old man with a synchronous metastatic hepatocellular carcinoma (HCC) and a metastatic well differentiated neuroendocrine tumor (Grade 2) of small bowel origin. The two histologies were biopsy proven from the sites of metastases (HCC—oral cavity, mesenteric, and cerebellar metastases; neuroendocrine—peritoneal metastases). From a systemic therapy viewpoint, he was treated with Sorafenib for the HCC and Sandostatin LAR for the neuroendocrine tumor. Around the time of initiation of systemic therapy, a large right ventricular mural‐based cardiac metastasis was noted on imaging. Although the cardiac metastasis was not biopsied, it was felt to be more likely HCC based on imaging characteristics. While he was asymptomatic and had good cardiac function, the decision was made to treat him with a course of radical palliative RT (54 Gy in 27 fractions) given a large size (6 cm) of cardiac tumor putting him at risk of cardiac compromise, and with the hope to reduce the size of disease and prevent its growth and associated morbidity. Volumetric modulated arc therapy (VMAT) technique with breath‐hold multiphase imaging was used to target the actual tumor and avoid as much of the rest of the heart as possible. Patient developed other unusual sites of metastases (oral cavity, cerebellum; both were successfully treated with palliative local therapies: RT and surgery, respectively). His cardiac condition was stable on last follow‐up, with no cardiac symptoms and preserved left ventricular function on echocardiogram; and up until 6 months after RT, CT imaging showed a decrease in the size of the cardiac metastasis. No further scans were carried out after the 6 months, as his condition deteriorated clinically from progression of his other HCC metastases, and he was not eligible for further systemic therapy. He died approximately 10 months after completing radiation to his heart.

*Patient 7* was a 53‐year‐old woman with stage 4 adenocarcinoma of the lung of the left upper lobe presented with minimal cardiorespiratory symptoms. CT imaging revealed multiple metastases, including a mural‐based mass in the left atrium and extending into pericardium. The cardiac mass had potential for impairing cardiac function, and therefore, a course of palliative RT (20 Gy in 5 fractions) to this area was given with the hope that it would defer any cardiac complications that may compromise her ability to undergo systemic therapy. She had stable clinical symptoms and cardiac mass until 3 months after radiation. Unfortunately, her lung disease progressed rapidly after and she developed significant dyspnea and multiple metastases. Patient opted for palliative care in a community unit.

### Palliative RT given as retreatment in a carefully selected patient

*Patient 8* was a 67‐year‐old woman with cardiac metastasis of thymic origin. She initially received 20 Gy in 5 fractions, and 39 Gy in 13 fractions 6 months later to achieve better response in the mediastinum. The tumor showed radiological response to RT, and she had stable disease for about 1 year until CT imaging showed a mural‐based cardiac metastasis with significant involvement of the right atrium and superior vena cava (SVC). She was mildly symptomatic from this mass. After discussion in multidisciplinary tumor board, she received RT targeted to the metastatic tumor area to avoid the development of SVC obstruction (SVCO) given that surgical resection was not feasible and she had excellent response to RT in the irradiated area. Because of overlap with the previous radiation field and in order to minimize any risk to the normal structures, RT was given as 30 Gy in 20 fractions twice a day. She tolerated treatment well with minimal side effects. A follow‐up CT scan 1 month after RT revealed an interval reduction in the size of tumor in the right atrium. She remained well with no clinical or radiologic evidence of cardiac tumor progression till 6 months after RT, when CT imaging showed an increase in the size of residual tumor and she became symptomatic with symptoms of SVCO. Her disease was not amenable to any treatment, and she went on SVC stent placement followed by referral to palliative care.

### Palliative RT for critically symptomatic CM may not be successful

*Patient 10* was a 66‐year‐old man with extensive metastatic rectal cancer and a large mass encroaching on the left atrium. He was quite symptomatic with fatigue, progressive shortness of breath, and chest wall pain. He was started on a course of palliative RT as 20 Gy in 5 fractions to cardiac tumor with the hope that radiation may decrease the volume of tumor and improve his symptoms. Patient declined hospital admission during the course of RT. He was near completion of treatment when his medical condition deteriorated with progressive congestive symptoms as a result of development of pericardial effusion and heart failure. Despite radiation treatment, the patient died after receiving 4/5 (16 Gy/20 Gy) planned RT.

### Palliative RT was delayed for tissue diagnosis

**Figure 1:**
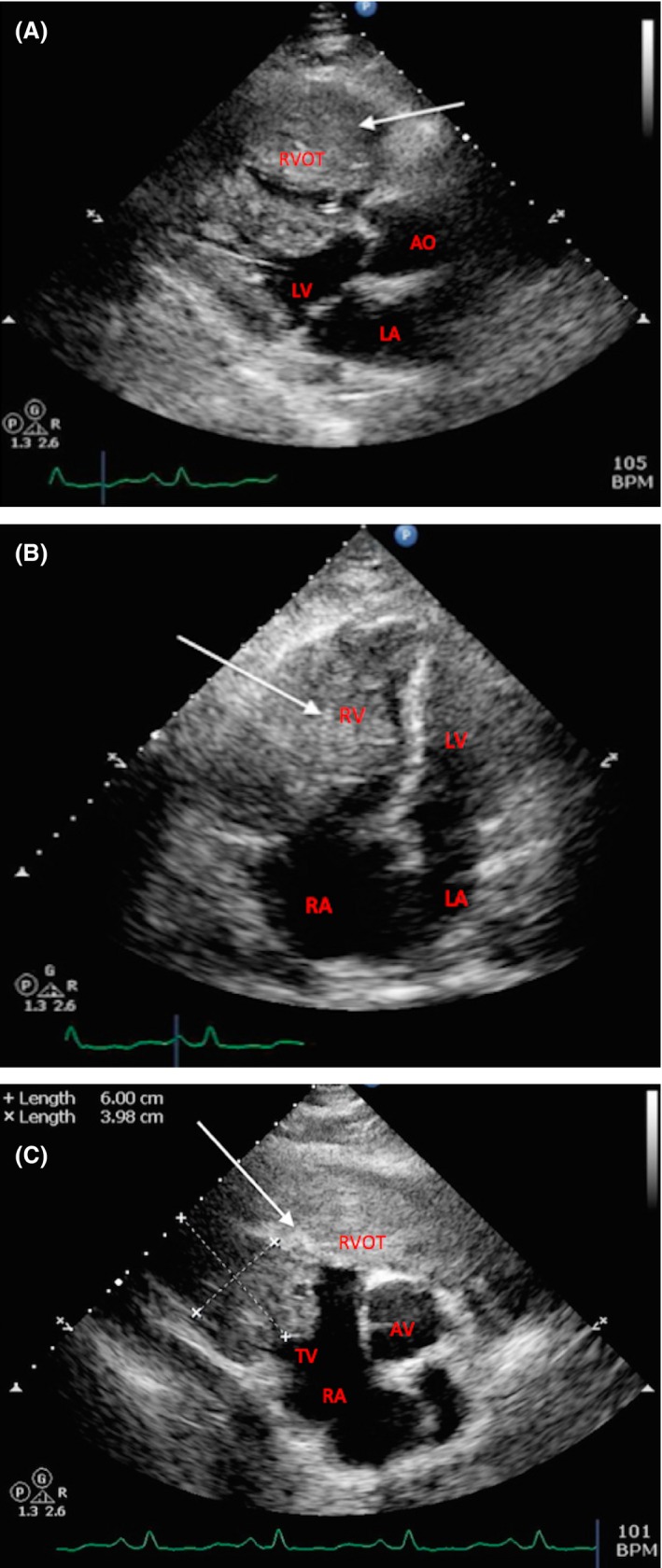
Transthoracic echo of patient 11. (A) Parasternal long‐axis view showing a small LV with normal function and a large 7.7 × 5.7 × 3.5 cm mass (arrow) causing an almost complete obstruction of RVOT, narrow pulse pressure, and low cardiac output; (B) apical 4 chamber view showing an obstructive RV mass (arrow) involving both septal and lateral walls of the LV and extending from base to apex; (C) short‐axis view at the level of AV showing RVOT mass (arrow) extending from the level of TV toward the pulmonic valve (RVOT, right ventricular outflow tract; AO, aorta; LV, left ventricle; LA, left atrium; RV, right ventricle; RA, right atrium; AV, aortic valve; TV, tricuspid valve).

*Patient 11* was a 62‐year‐old man presented to emergency department with progressive shortness of breath and pleuritic chest pain since 2 months prior to admission. He was admitted to cardiac care unit and further workup showed extensive metastatic disease including a soft‐tissue mass within the right ventricle. Initial clinical impression was that the cardiac mass may be the primary tumor as it was very large. He was not a candidate for surgical resection because of the extent of cardiac involvement and advanced stage of disease. Tissue diagnosis was pursued with the hope to start the patient on appropriate systemic treatment; palliative RT was considered but was held awaiting tissue diagnosis. Intra‐cardiac echo‐guided biopsy of tumor mass was performed, but results were inconclusive. The patient had coronary angiography showing tumor blush where a large leash of vessels from right coronary artery supplied the tumor. Figure 1 shows an interesting observation that the cardiac mass had caused the complete right ventricular outflow tract (RVOT) obstruction. A subsequent biopsy from one of the soft‐tissue masses (buttock/abdomen) was inconclusive. Unfortunately, the patient died 1 week after admission, without receiving any treatment. Autopsy revealed that the cardiac and all other tumors were malignant melanoma.

## Discussion

The majority of cardiac tumors are metastatic, with the most common source being melanoma and mediastinal primary tumors 1. Cardiac metastasis should be suspected in a cancer patient with disseminated disease who has a sudden onset of unexplained respiratory symptoms, chest pain, tachycardia, arrhythmia, cardiomegaly, or heart failure 1, 9. Metastasis to the heart occurs in approximately 10% of patients with disseminated malignancies 1, 2, and melanoma is commonly referred to as the neoplasm with the highest rate of metastases to the heart 1, 10. Endomyocardial biopsy is a commonly performed procedure to aid in the diagnosis of cardiac masses 11. It is especially reasonable to undertake for right‐sided cardiac masses showing infiltration or obstruction 5, 12, and if the diagnosis cannot be established by noninvasive modalities or less invasive noncardiac biopsy, or if tissue diagnosis is expected to influence the course of therapy 11.

Patients with CM are incurable and usually have disseminated disease, but still an appropriate medical management and aggressive multidisciplinary treatment may improve quality of life and lengthen patient's survival 5, 8. A multimodality approach with surgery in selected patients, chemotherapy, and palliative RT may provide long‐term remission and an improvement in survival 5, 8. The role of palliative RT in managing CM has been somewhat under‐appreciated because of the poor prognosis of patients and paucity of literature to guide treatment decisions. To our knowledge, our report includes the largest number of patients with mural‐based CM treated in the modern era of radiation therapy using modern CT simulator and three‐dimensional (3D) imaging. This report is also unique as it outlines the role of palliative RT in patients with primary histologies that have not previously been reported in the literature (e.g., cases with primary HCC and rectal cancer). The provided summary of available literature (Table 1), along with the clinical scenarios described here can be used as educational aids for radiation oncology practice and training.

Patients with CM have generally poor prognosis, and considering the risks and complications of the heart surgery, it is important to carefully select the patients for surgical resection, probably with a rather restrictive approach 11. Surgical resection of CM is usually reserved for exceptional patients with good prognosis, cases of intracardiac obstruction, and for cases where complete or near‐complete resection is feasible 4, 12, 13. Few studies report urgent indications for surgical interventions, including acute symptoms such as pulmonary or systemic embolism and cardiogenic shock due to obstructive tumor 9, 11. If feasible, resection of CM offers the chance of maximum local control. In these highly selected patients, postoperative RT and/or chemotherapy is a reasonable consideration to reduce the chance of local recurrence. Based on the beneficial experience with adjuvant RT for soft‐tissue sarcoma at other sites and anecdotal evidence that adjuvant RT can provide a local control (and perhaps survival) benefit in primary cardiac sarcomas 1, 14, 15, 16, 17 postoperative RT in carefully selected cases with CM can yield good local tumor control and improve the durability of symptom palliation 1, 2.

Many patients with CM are not good candidates for surgery or chemotherapy due to tumor unresectability, poor general condition, and the issue that systemic therapy is not likely to yield a significant rapid response. For this reason and because radiotherapy is potentially effective in most of the tumors (regardless of histology), it is reasonable to consider palliative RT in some patients, based on clinical and imaging findings without tissue diagnosis. A delay in palliative RT in severely symptomatic patients, either to obtain tissue diagnosis in case of undiagnosed primary tumor or to initiate chemotherapy, could potentially deprive these patients of an effective treatment that can provide symptom control and has a reported response rate of up to 60% 9. Such delays could potentially be fatal as a result of catastrophic embolization and/or cardiopulmonary decompensation from progressively enlarging and infiltrating tumors causing blood flow obstruction. Despite their complicated medical condition, when offered early in the course of disease, our patients tolerated RT well. Considering the beneficial effect of palliative RT for patients reported here and elsewhere (Table 1), we recommend that irradiation of CM should be considered early in the management of these patients, particularly when other treatment options are not feasible or do not seem promising. Moreover, in carefully selected cases, palliative RT can be delivered while a patient receiving chemotherapy targeting the primary tumor 10, 18.

The optimal radiotherapy dose and fractionation should be individualized based on patient‐ (age, performance status, prognosis, and clinical symptoms), tumor‐ (histology, size, location, and extent of systemic disease), and treatment‐related (history of previous thoracic RT, other therapeutic options) factors and on case by case basis. The currently available data on primary cardiac sarcomas suggest that restricting the postoperative RT dose to 45–50 Gy will minimize the risk of radiation‐induced toxicity 1. It appears that the total radiation dose of 30–45 Gy in 1.8–2 Gy per fraction is safe, effective, and desirable to provide symptom control in patients with CM (Table 1). Cham et al. 9 reported their experience with radiotherapy of 38 patients with cardiac and pericardial metastases treated in the pre‐3D planning era from 1952 to 1971 using 250 kVp equipment, old radiotherapy techniques, and clinical setup and portal films. Most patients received 25–35 Gy in 1.5–2 Gy per fraction in 3–4 weeks. While radioresistant tumors may need higher radiation dose, low‐ to moderate‐dose palliative radiotherapy can provide reasonable symptomatic relief in radioresponsive tumors, with a low risk of acute toxicity. We recommend the short‐course hypofractionated schedules with large dose per fraction (30 Gy in 10 fractions, 20 Gy in 5 fractions, or single 8 Gy) to minimize patients' time on treatment given their short life expectancy, the convenience of treatment, lower costs, and the need to start chemotherapy regimens in a timely fashion. The current advances in imaging technology and computer‐guided RT planning techniques may allow delivery of higher radiation doses and re‐irradiation, when debulking of tumor is desired. For instance, using intensity‐modulated radiation therapy (IMRT) technique and contouring noninvolved cardiac structures as avoidance structures, we can deliver higher and potentially more effective RT doses to the tumor while minimizing the dose to uninvolved parts of the heart and lungs. Dasgupta et al. 10 has reported a successful treatment of a patient with cardiac metastasis involving the pacemaker leads with careful consideration of radiotherapy plan, pace maker interrogation before and after daily RT, and close cardiac monitoring during RT. Patients with CM often have positional dyspnea and may not tolerate the supine position for a long treatment delivery. New treatment techniques like VMAT have the advantage of a shortened delivery time, which improves patient comfort during treatment.

Heart irradiation can cause various early and late side effects; coronary artery disease, pericarditis, myocarditis, valvular damage, arteritis, ventricular systolic or diastolic dysfunction, and disturbance of the conduction system 19. Pericarditis is the most common early toxicity presenting within 2–6 months post‐RT. Radiation treatment may facilitate tumor thrombosis and cause blood flow obstruction. With large fraction sizes, there is also a concern about abrupt tumor death resulting in an arterial embolus of tumor or rupture of vessel wall 20. The presence of cardiac dysfunction, arrhythmias, and pace maker in these patients could further complicate the radiation treatment 10. The late radiation side effects are of less concern in patients with CM due to short survival in these patients.

## Conclusion

Metastasis to the heart remains a treatment challenge, with a dismal prognosis despite all available treatments. The optimal treatment approach and sequence of therapies must be individualized after discussion in multidisciplinary team. The success of palliative RT in improving clinical symptoms and achieving good local control calls for further consideration of this modality either as a single modality or in combination with surgery and/or chemotherapy. Palliative RT should be considered early in the management of patients with CM and should not be a treatment of last resort, which has been the tradition in the past.

## Conflict of Interest

None declared.
